# The piecewise parabolic method for elastic-plastic flow in solids

**DOI:** 10.1038/s41598-018-28182-7

**Published:** 2018-07-03

**Authors:** Wei Zhang, Cheng Chen, Kun Liu, Jing-Song Bai, Ping Li, Zhen-Hua Wan, De-Jun Sun

**Affiliations:** 1 Department of Modern Mechanics, University of Science and Technology of China, Hefei, 230027 China; 2 Institute of Fluid Physics, China Academy of Engineering Physics, Mianyang, 621900 China; 3 Low Speed Aerodynamics Institute, China Aerodynamics Research & Development Center, Mianyang, 622762 China; 4 Shock and Vibration of Engineering Materials and Structures Key Laboratory of Sichuan Province, Southwest University of Science and Technology, Mianyang, 621010 China

## Abstract

A numerical technique of high-order piecewise parabolic method in combination of HLLD (”D” denotes Discontinuities) Riemann solver is developed for the numerical simulation of elastic-plastic flow. The introduction of the plastic effect is realized by decomposing the total deformation gradient tensor as the product of elastic and plastic deformation gradient tensors and adding plastic source term to the conservation law model equation with the variable of the elastic deformation gradient tensor. For the solution of the resulting inhomogeneous equation system, a temporal splitting strategy is adopted and a semi-implicit scheme is performed to solve the ODES in the plastic step, which is conducted to account for the contributions from plastic source terms. As seen from the results of test cases involving large deformation and high strain rate, the computational model used can reflect the characteristics of constitutive relation of material under strong impact action and our numerical method can realize the exact simulation of the elastic-plastic behavior of solid material, especially the accurate capture of the elastic-plastic waves. Further, it could also deal with high-speed impact problems with multi-material components, catching material interfaces correctly and keeping the interfaces sharp, when combined with interface tracking technique such as the level-set algorithm.

## Introduction

The elastic-plastic flow problem of solid material under high-load conditions are often encountered in martial and industrial applications. As it is featured with strong nonlinearity of the interactions between different materials under the extreme conditions such as high temperature, high pressure and high speed, the relevant theoretical and experimental studies have received relatively large difficulties while numerical computation is natural to become the main research means because of the obvious advantages such as flexible executive way, controllable process, high efficiency-cost ratio and feasibility to complex geometries and load status.

Among all numerical methods for the elastic-plastic flow problem, the Lagrangian method is widely applied in engineering area^1–3^, since it is not necessary to process material interface for this view because the mesh point and the material point are always in agreement during the deformation process of material, although there exists some issues for the treatment of plastic problems with large deformation. Compared with Lagrangian method, Eulerian method^4–6^ is more applicable to the problems with large material deformation since mesh warp or distortion induced could be largely avoided in the fixed mesh framework, although it is a great challenge to apply the Eulerian method to capture material interfaces therein. In the following, we formulate the elastic-plastic flow problem with full Eulerian method, which is considered to have advantages for capturing shocks naturally and providing a smooth transition between solids and fluids in the case of phase change.

A variety of models in the Eulerian frame in recent years have been developed to study elastic-plastic flow of solid material. As for plasticity behavior, its modelling is still an open issue. Particularly, the introduction of plasticity in the relevant partial differential equations (PDE) models as well as its definition in the case of large deformation lacks clarity and uniqueness^7,8^. Nevertheless, plasticity models are all developed from either hypoelastic or hyperelastic models in any case. Since several problematic points in the plastic behavior models based on the former, which are widely used in engineering area with the stress components as equation variables^2,9,10^, need to be investigated further^8^, several researchers had conducted the investigations of plastic behavior model based on the latter. In view that constitutive relation equation for stress of material satisfies Von Mises yield criteria, Kluth^11^ introduced a modified equation of state to the control equation for hyperelastic model, realizing the description of plastic behavior of the material under impact action. However, this description way is only applicable to perfect plasticity and has some difficulty in the achievement of the reflection of elastic-plastic shock wave on free interfaces. Another way to depict plastic behavior based on hyperelastic model is to decompose the total deformation tensor (deformation gradient tensor or its inverse) into the two parts of elastic and plastic deformation by using multiplicative decomposition method and to realize the characterization of plastic effect by means of the additional source term. Plohr^12^ had constructed both rate-independent and rate-dependent plasticity model based on the inverse of deformation gradient tensor and obtained a larger equation system with the total equation number of 21. And, Miller^13^ and Barton^14^ had taken the second way, i.e. added the plastic source term to the control equation for elasticity based on deformation gradient tensor, to realize the depiction of plastic effect.

Considering that elastic-plastic shock wave and discontinuousness exist in the elastic-plastic flow problem widely and the numerical method of Godunov type has been proven to have apparent superiority for capturing both shock wave and discontinuousness in fluid mechanics, it is natural to extend the use of high-order scheme of Godunov type based on the solution of local Riemann problem to solid mechanics in Eulerian framework, in case that the control equations are constructed to be of properly conservative form. Accordingly, several schemes of Godunov type, such as piecewise linear scheme, first-order Godunov scheme, MUSCL-Hancock scheme and MUSCL scheme, have been applied in the elastic-plastic deformation problems (see Miller *et al*.^13^; Kluth *et al*.^11^; Hank *et al*.^15^; Favrie *et al*.^16^; Howell *et al*.^1^; Brauer *et al*.^4^). Note that the above works are mostly focused on modifying the piecewise-constant distribution of original variables as the piecewise-linear distribution in space, achieving second-order accuracy of numerical computation. To our knowledge, the numerical scheme of Godunov type with higher order accuracy, such as Piecewise Parabolic Method (PPM)^17^ which has third-order accuracy and stronger ability of processing discontinuousness, has not been utilized in the problem of elastic-plastic deformation.

In addition to scheme precision, the solution of local Riemann problem is another important and noticeable respect for the schemes of Godunov type. In the nonlinear hyperbolic system of fluid mechanics, it is commonly realized by linearizing the Jacobian matrix, e.g. Roe scheme^18^. While for solid mechanics, it is considerably difficult to derive the linearized Jacobian matrix analytically due to the complexity of equation of state. Thus, two kinds of numerical method for the computational treatment of this problem are raised. The first choice is to do the arithmetical average of Jacobian matrices at the left and right cell centre states simply^19^. However, this way failed to solve the Riemann problem when applied in the impact case of nonlinear elasticity^19^. The second choice is to make use of Harten-Lax-van Leer (HLL) solver based on two-wave assumption^20^ and HLLC solver (‘C’ denotes Contact) based on three-wave assumption^21,22^. Howell *et al*.^1^ had used HLL solver to simulate two-dimensional elastic-perfectly plastic deformation problem of solid material and the application of HLLC solver in the elastic-plastic deformation problem had been discussed in Ndanou *et al*.^23^, Cheng *et al*.^24^ and Brauer *et al*.^4^. However, HLL and HLLC solvers based on two-wave and three-wave assumptions may be inadequate when applied to nonlinear elasticity, in view that the exact wave pattern of Riemann problem may involve more than five distinct waves therein. Thus, we have developed HLLD (‘D’ denotes Discontinuities) solver based five-wave assumption and found that it has obvious advantages in capturing wave patterns containing more than five waves when applied to nonlinear elasticity (see Zhang *et al*.^25^. In this paper, the numerical technique of PPM reconstruction combined with HLLD solver is chosen to simulate the elastic-plastic deformation behavior of solid materials, with the purpose of both the derivation of high order numerical accuracy and the accurate capture of multi-wave patterns.

## Results

### Test case 1: Accuracy test

To test the accuracy of our method in a plasticity-dominated problem, we perform the computation for a one-dimensional nontrivial and shockless problem similar to the purely elastic problem presented in Zhang^25^. Two initially Gaussian distributions with width 5 are used to initialize *F*_22_ and *F*_33_ as functions of coordinate *x*_*i*_:

1 $${F}_{\mathrm{11,}i}=\mathrm{1/1.1,}\,{F}_{\mathrm{22,}i}=\mathrm{1/[1.1(1}+9{\omega }_{i})],{F}_{\mathrm{33,}i}=\mathrm{(1}+9{\omega }_{i}\mathrm{)/1.1,}\,{\omega }_{i}=\mathrm{1/}a/\sqrt{2\pi }\,\exp (\,-\,{x}_{i}^{2}\mathrm{/(2}{a}^{2}\mathrm{)).}$$

The other components of velocity u and F are all set to 0. The parameters for equation of state 15 and constitutive model are as follows: *ρ*_0_ = 2.7 *g/cm*^3^, *p*_01_ = 73 *GPa*, *p*_02_ = 172 *GPa*, *p*_03_ = 40 *GPa*, *μ* = 24.8 *GPa*, *σ*_*y*_ = 0.2976 *GPa*, *τ*_0_ = 1.

**Figure 1:**
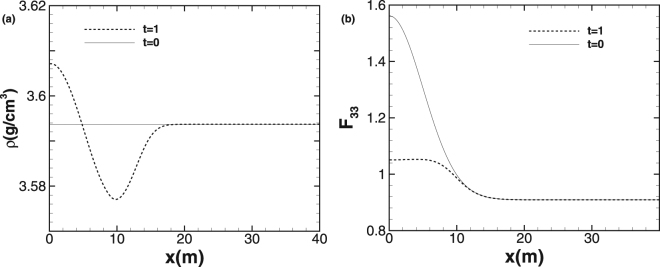
The initial conditions and computed results of the accuracy test problem. (**a**) The profiles of density *ρ*; (**b**) deformation gradient *F*_33_.

The computational region is 0 *m* ≤ *x* ≤ 40 *m* and a transmissive boundary condition is used. We consider the solution derived by PPM + HLLD scheme with a fine mesh as the reference solution for accuracy and error analysis. Here, the grid spacing is 0.1 *m* and the CFL number is set to be 0.1 so that the dominant error is the spatial error. Figure 1 plots the initial conditions and computed results of this test case at *t* = 1 *ms*. In this scale, the differences between the results at 50, 100, and 200 grid points are nearly invisible. The convergence rate of PPM + HLLD scheme are presented in Table 1. From Table 1, we could conclude that the numerical results converge with the order of 3 and thus the PPM + HLLD scheme is third-order accurate for smooth problems.

**Table 1:** *L*_1_ Errors and orders of convergence for plasticity-dominated smooth problem.

Scheme	N	*ρ*		*u*_1_		*F*_11_	
		*L*_1_-error	*L*_1_-order	*L*_1_-error	*L*_1_-order	*L*_1_-error	*L*_1_-order
PPM + HLLD	50	3.22E-04	—	1.84E-04	—	2.74E-05	—
	100	5.78E-05	2.48	3.20E-05	2.53	4.72E-06	2.54
	200	7.61E-06	2.93	4.46E-06	2.84	5.82E-07	3.02

### Test case 2: Elastic-plastic piston-like problem

This case, from Maire *et al*.^26^, is similar to the one-dimensional hydrodynamic piston test. At the initial time, the copper medium with the length interval of 0 *m* ≤ *x* ≤ 1 *m* is at rest. The velocity at its left boundary is *u*_1_ = 20 *m/s* and its right boundary satisfies transmissive boundary condition. The material parameters for copper medium are: *ρ*_0_ = 8.93 *g/cm*^3^, *p*_01_ = 140.7 *GPa*, *p*_02_ = 287.1 *GPa*, *p*_03_ = 233.5 *GPa*, *μ* = 45 *GPa*, *σ*_y_ = 0.09 *GPa*. The analytical solution of this problem consists of a plastic shock wave and an elastic precursor shock wave. This test case is run until the termination time *t* = 0.15 *ms*.

**Figure 2:**
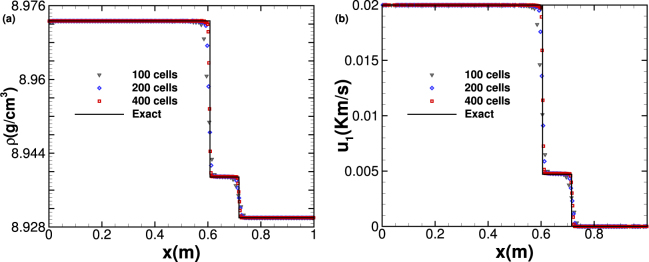
Density and velocity resulting from the elastic-plastic piston-like problem at time *t* = 0.15 *ms*.

The convergence of the numerical results derived by the PPM + HLLD method with meshes of 100, 200 and 400 cells is presented in Fig. 2. As seen from Fig. 2, the results with different grid resolutions are all converged to the exact solution from Maire *et al*.^26^ and our numerical method could capture elastic and plastic shock waves effectively without numerical oscillation at the locations of shock waves. Compared with the results of Maire *et al*. derived by second-order cell-centered Lagrangian scheme, our results determined by PPM + HLLD are closer to the exact solution with the same grid resolution. The *L*_1_-norm errors and convergence rates of density ρ and velocity *u*_1_ as well as CPU times taken for different grid numbers are presented in Table 2. The numerical accuracy of the whole scheme is first order due to the existence of shock waves in this case.

**Table 2:** *L*_1_-norm errors and convergence rates as well as relative CPU times for elastic-plastic piston-like problem.

N	*ρ*		*u*_1_		Relative CPU time
	*L*_1_-error	*L*_1_-order	*L*_1_-error	*L*_1_-order	
100	4.66E-04	—	2.14E-04	—	1
200	2.47E-04	0.92	1.13E-04	0.92	3.8
400	1.26E-04	0.97	5.82E-05	0.96	10.6

### Test case 3: Elastic-plastic impact problem

This test case, similar to the impact test problem in Titarev *et al*.^19^, describes the impact situation of two semi-infinite slabs of aluminum traveling with the same velocity and in opposite directions. The computational region is 0 *m* ≤ x ≤ 10 *m* and the initial discontinuousness lies at the location of *x*_0_ = 5 *m*. Further, the parameters for the state equation and constitutive model of aluminum medium are as follows: *ρ*_0_ = 2.7 *g/cm*^3^, *p*_01_ = 73 *GPa*, *p*_02_ = 172 *GPa*, p_03_ = 40 *GPa*, *μ* = 24.8 *GPa*, *σ*_*y*_ = 0.2976 *GPa*, *τ*_0_ = 10^−9^ *s*.

**Figure 3:**
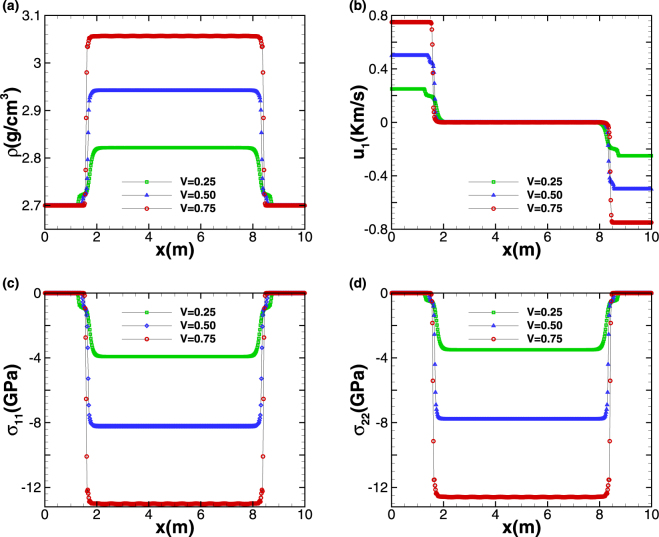
The distributions of density ρ (**a**), the velocity in the *x* direction *u*_1_ (**b**) as well as the stress components σ_11_ (**c**) and σ_22_ (**d**) at *t* = 0.6 *ms* with different impact speeds, the velocity unit is km/s.

Figure 3 gives the distributions of density *ρ*, the velocity in the *x* direction *u*_1_ as well as the stress components *σ*_11_ and *σ*_22_ at *t* = 0.6 *ms* with different impact speeds. The results imply that with lower impact speed, the aluminum material is of elastic-plastic nature and the symmetric impact of aluminum slabs generates symmetric elastic-plastic shock waves. While as stated in Titarev *et al*.^19^, the aluminum material is elasticity and only the symmetric elastic waves arise in the resulting wave structures. With further increase of impact speed, both the intensity of shock wave and plastic effect are enhanced, and the stress and density behind the shock wave become larger. As PPM + HLLD uses flattening algorithm to add a little dissipation at the locations of strong shock waves, which is introduced by an additional slope limiter via a ‘flattening’ parameter and suppresses spurious post-shock oscillations for strong shock waves, and there is no non-physical undershoots and overshoots as in the computations of Titarev *et al*.^19^, our numerical method is shown by this case to have good effectiveness for solving high speed impact problem.

### Test case 4: Wilkins’s problem

This test was originally proposed by Wilkins^10^ and deals with the one-dimensional impact of a thin aluminum flyer into a half space of aluminum plate. The initial one-dimensional computational region 0 *mm* ≤ *x* ≤ 50 *mm* consists of two states which are separated by an interface at *x*_0_ = 5 *mm*. The left state is moving with a non-zero velocity while the right state is initially at rest. The materials of the flyer and plate considered are both aluminium, and other material parameters are the same as in the test case 3. At the right boundary of aluminum plate, the transmissive boundary condition is satisfied. The left side of flyer is surrounded by vacuum. Further, the interface location is tracked via the level set field and a solid-vacuum Riemann problem is solved to determine the values at the ghost cells at the left boundary.

**Figure 4:**
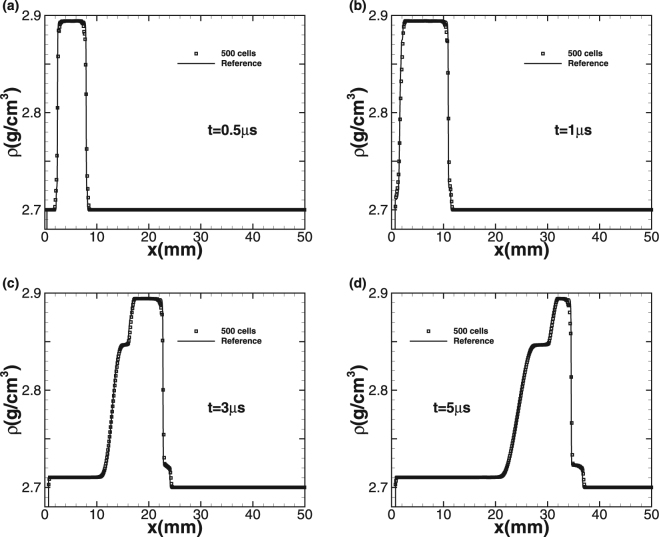
Time sequence of density *ρ* for Wilkins’s problem with impact speed 0.8 km/s.

**Figure 5:**
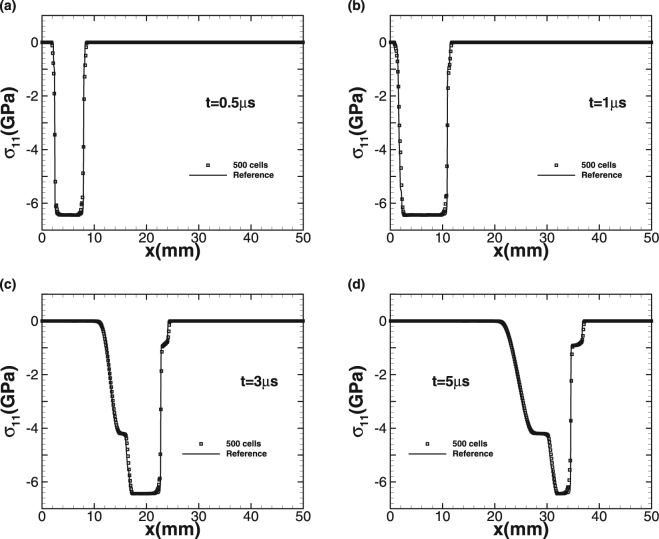
Time sequence of normal stress σ_11_ for Wilkins’s problem with impact speed 0.8 km/s.

**Figure 6:**
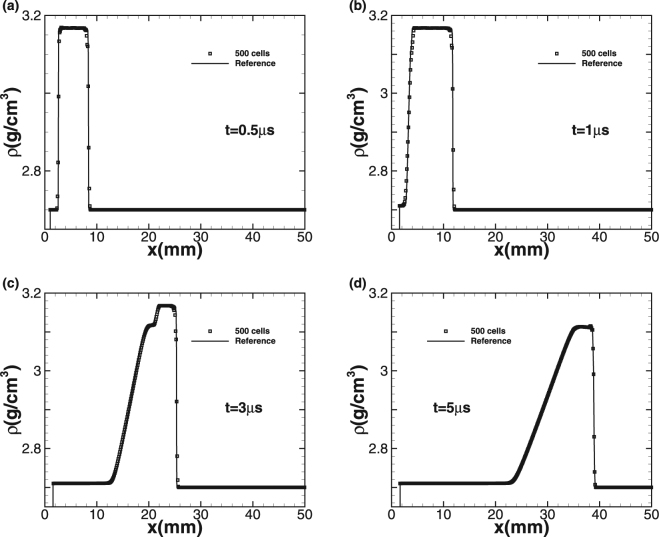
Time sequence of density *ρ* for Wilkins’s problem with impact speed 2 km/s.

**Figure 7:**
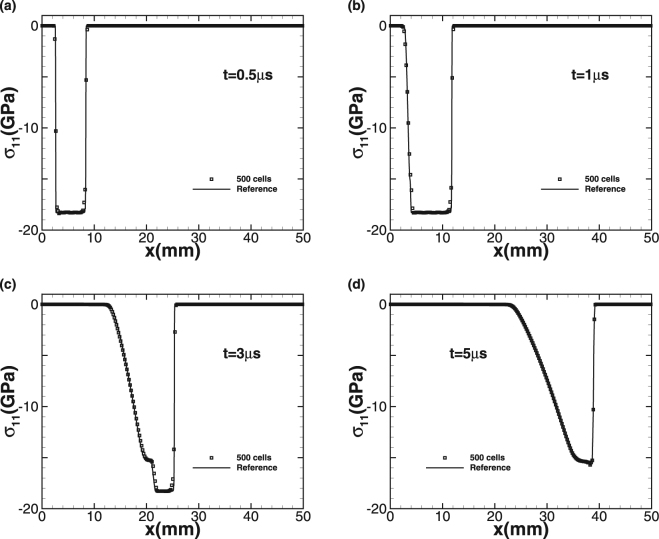
Time sequence of normal stress σ_11_ for Wilkins’s problem with impact speed 2 km/s.

The simulation starts at the moment that the flyer plate is just in touch with the target. We use 500 cells to simulate the problem up to the termination time *t* = 5 *μs* and CFL is equal to 0.6. Since there is no exact solution for comparison, we generate a reference solution by running a high-order method on fine-mesh. Two impact speeds *u*_1_ = 0.8 *km/s* and 2 *km/s* are considered. The plots of density *ρ* and normal stress σ_11_ at different times are given with the impact speed *u*_1_ = 0.8 *km/s* in Figs 4 and 5, respectively. Squares indicate numerical solution while the solid line represents the fine-mesh reference solution. For the lower impact speed, the impact of the aluminum flyer into the plate results in two elastic-plastic shock waves travelling to both the left and right sides. With increasing time, the right-travelling elastic shock wave is distinguishable from the right-travelling plastic shock wave in the right-travelling shock waves due to the larger speed of elastic wave, forming speed and stress steps. The left-travelling shock wave reaches the left free surface and is subsequently reflected to form a right-travelling rarefaction wave. Afterwards, this rarefaction wave is split into elastic rarefaction wave and plastic rarefaction wave obviously due to different wave speeds, which is the so-called elasticity precursor phenomena, and the right-travelling rarefaction wave begins to pursue the initial right-travelling waves. When larger impact speed *u*_1_ = 2 *km/s* is imposed, the plastic phenomena is more obvious and the peak values of density and stress become even larger, as shown in Figs 6 and 7. As the elastic limit has been exceeded at this time, only the left-travelling and right-travelling plastic shock waves are generated after the impact event. Subsequently the left-travelling plastic shock wave reaches the left free surface and is reflected to form right-travelling rarefaction wave, which will be split into elastic rarefaction wave and plastic rarefaction wave some time later and catch the initial right-travelling plastic shock wave eventually. As no analytic solution exists for this problem leading to incapability of quantitative comparison analysis, but our results of the peak values of both wavespeed and stress are seen to be in good qualitative agreement with wilkins *et al*.^10^. Further, this case approves both the ability of our numerical technique on processing multi material interfaces and its strong robustness for the plastic-dominated case.

### Test case 5: Two-dimensional impact

**Figure 8:**
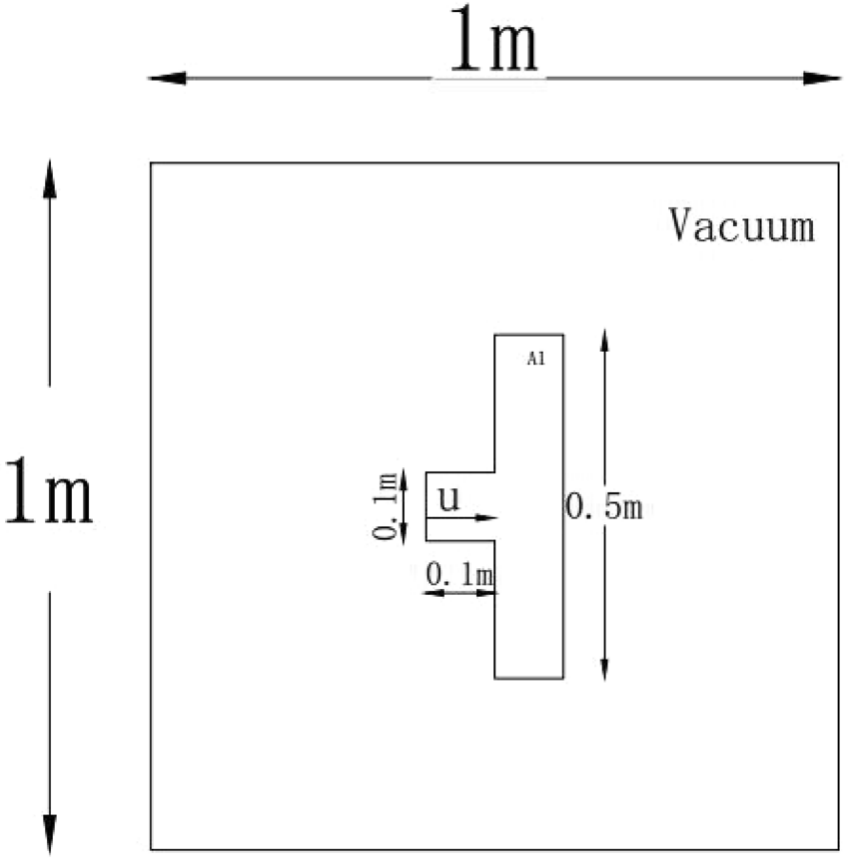
Schematic of initial conditions for the two-dimensional impact case.

In this two-dimensional case, we utilize the PPM + HLLD scheme for the multi-material situation to investigate the impact problem of a projectile on a solid plate surrounded by vacuum. The initial configuration of the impact problem is depicted in Fig. 8, in which the projectile is a square with sides of length 0.1 *m* and the plate is 0.5 *m* long and 0.1 *m* wide. The materials of the projectile and plate are assumed to be aluminium. At *t* = 0, the projectile gets in touch with the plate and all materials are assumed to be in a stress free state: F = I and S = 0. Further, the aluminium target is set to be static, while the aluminium projectile is initialized with *u*_1_ = 800 *m/s*. The computational domain is [−0.5 *m* ≤ *x* ≤ 0.5 *m*, −0.5 ≤ *y* ≤ 0.5 *m*]. The mesh sizes are set as Δ*x* = Δ*y* = 1/800 *m*, and the CFL number is fixed to be 0.4.

**Figure 9:**
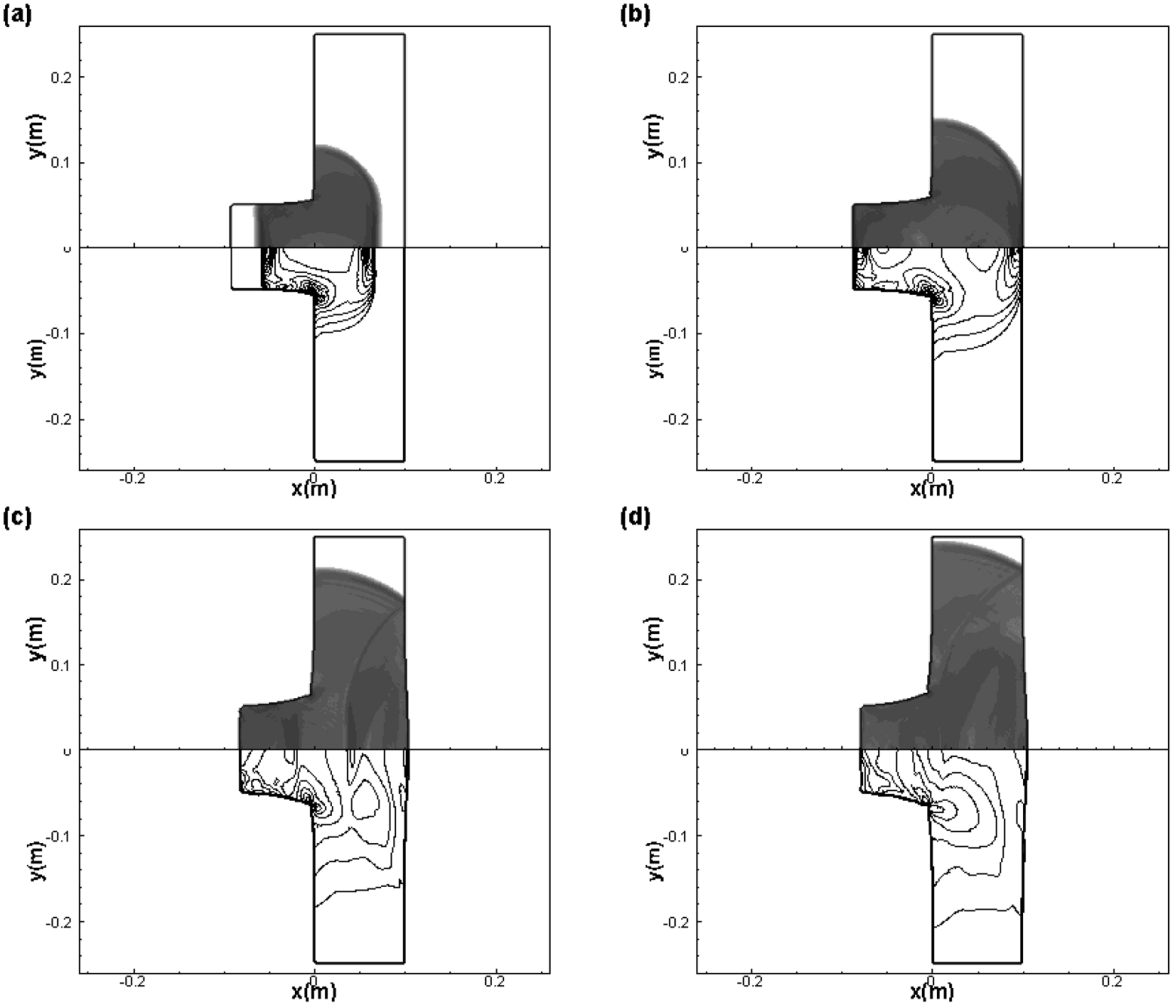
Numerical Schlieren pictures of the density (top) and the von Mises criteria ${|\sigma ^{\prime} |}^{2}-\frac{2}{3}{({\sigma }_{y})}^{2}$ (bottom) at *t* = 1.0 × 10^−5^ *s* (**a**), *t* = 1.5 × 10^−5^ *s* (**b**), *t* = 2.5 × 10^−5^ *s* (**c**) and *t* = 3.0 × 10^−5^ *s* (**d**) for the two-dimensional impact case, *u*_1_ = 800 *m/s*.

Figure 9 gives the density schlieren (top) and the von Mises criteria ${|\sigma ^{\prime} |}^{2}-\frac{2}{3}{({\sigma }_{y})}^{2}$ (bottom) for times *t* = 1.0 × 10^−5^ *s*, *t* = 1.5 × 10^−5^ *s*, *t* = 2.5 × 10^−5^ *s* and *t* = 3.0 × 10^−5^ *s*. These schlieren images are all obtained by plotting |▽ρ| field using a logarithmic scale. As seen from Fig. 9(a), the longitudinal shock wave and transverse shear wave are generated in the impactor and plate after the impact. As the speed of the latter, which induces plastic deformation, is lower than that of the former, the wave front of von Mises criteria is slower than that of the longitudinal shock wave in the density plot. With increasing time, both waves spread to the right boundary of plate and are reflected back to the plate (Fig. 9(b,c)). Accompanied with the propagation of waves in the plate and their multiple reflections, the deformation of the plate is observed clearly and a bulge is formed at the back part. Further, the plastic effects are quite obvious at both the location of contact and the back part of plate, where large deformation exists, whereas other parts bear a relatively small plastic strain. The robustness of the technique PPM + HLLD in two-dimensional cases is well verified based on these qualitative results.

## Discussion

This paper has constructed the PPM method suitable for the elastic-plastic deformation problem of solid material under the impact action. This method is based on the elastic-plastic model equation under Eulerian framework, which is formed by the addition of plastic source term into the elastic model equation with deformation gradient tensor as the original variables. In our previous work, the HLL-type solvers are applied to the Riemann problem of nonlinear elasticity and HLLD Riemann solver is shown to be more effective compared with HLL and HLLC solvers for the problems with solution structure of more than five waves. Furthermore, the numerical technique of PPM combined with HLLD solver is seen to have strong accuracy and reliability for the solution of Riemann problem. For the elastic-plastic problem in this study, the solution way is to apply the splitting method to decompose the elastic-plastic problem into the solutions of elasticity control equation and plastic source term. With the purpose of extending the numerical technique of PPM + HLLD to the elastic-plastic problem, the difficulty lies in the fact that the plastic source term may range from zero to positive infinity (i.e. the nature of solid material changes from elasticity to fluid), leading to very small time scale with potentially quite high strain rate. To prevent the possible numerical rigidity, we use the semi implicit method, which is of less computational cost and easier to be realized compared with full implicit method, to solve the ODES for the plastic source term. The high order and effectiveness of our method are confirmed by one-dimensional nontrivial and shockless problem (test case 1) and elastic-plastic piston-like problem (test case 2), respectively. For solving the impact problem with multi solid materials involving different material interfaces and even the interface between solid material and vacuum, we have applied the modified ghost fluid method in fluid mechanics, where the level-set method is used to track material interface and the interaction of the different materials at the two sides of the interface is simulated by the HLLD Riemann solver for multi material cases. The effectiveness of the above method is fully reflected by the computational results of Wilkins’s problem and two-dimensional elastic-plastic impact problem.

## Methods

### Governing equations

We follow the formulation presented in Zhang^25^, where the model equations proposed by Godunov & Romenski^27,28^ are used in Eulerian framework for nonlinear elasticity, and extend it to plasticity modelling, so as to accomplish the study of elastic-plastic deformation behavior of solid materials. The hyperbolic partial differential equations that depict mass, momentum and energy conservation in Cartesian coordinates read

2 $$\frac{\partial \rho }{\partial t}+\frac{\partial \rho {u}_{k}}{\partial {x}_{k}}=0$$

3 $$\frac{\partial \rho {u}_{i}}{\partial t}+\frac{\partial (\rho {u}_{i}{u}_{k}-{\sigma }_{ik})}{\partial {x}_{k}}=0$$

4 $$\frac{\partial \rho E}{\partial t}+\frac{\partial (\rho {u}_{k}{\rm{E}}-{u}_{i}{\sigma }_{ik})}{\partial {x}_{k}}=0$$

where *ρ* denotes the material density, *u*_*i*_ denotes velocity, σ is the Cauchy stress, *E* = *ε* + *u*_*i*_*u*_*i*_/2 is the total energy, *ε* is the specific internal energy and the Einstein summation convention over repeated indices is implied (*i, j, k* = 1, 2, 3).

The strain equation of nonlinear elasticity is described in terms of deformation gradient tensor *F*_*ij*_ = ∂*x*_*i*_/∂*X*_*j*_ (*x*_*i*_ and *X*_*j*_ represent the fixed Eulerian coordinates and Lagrangian coordinates respectively):

5 $$\frac{\partial \rho {F}_{ij}}{\partial t}+\frac{\partial (\rho {F}_{ij}{u}_{k}-\rho {F}_{kj}{u}_{i})}{\partial {x}_{k}}=-\,{u}_{i}{\beta }_{j}\mathrm{.}$$

Here ${\beta }_{j}=\frac{\partial \rho {F}_{kj}}{\partial {x}_{k}}$ is an artificial vector variable that provides the conservative form of equations for *F*_*ij*_. In order to capture the correct wave speed in the quasi-linear system (12) and to ensure the correctness of numerical results, the term −*u*_*i*_*β*_*j*_ is treated as a source term in the numerical computation. To extend elastic strain equation to account for plasticity, we utilize a multiplicative decomposition of the total deformation gradient tensor^13^

6 $${F}^{T}={F}^{e}{F}^{p},$$

where the superscript ‘*e*’ denotes elastic and ‘*p*’ means plasticity. The evolution of *F*^*p*^, the plastic part of deformation gradient tensor, satisfies the general expression

7 $$\frac{D{F}^{p}}{Dt}={L}^{p}{F}^{p},$$

where *L*^*p*^ is called the plastic deformation rate tensor. After substituting (6,7) into (5), the following formula can be obtained:

8 $$\frac{\partial \rho {F}_{ij}}{\partial t}+\frac{\partial (\rho {F}_{ij}{u}_{k}-\rho {F}_{kj}{u}_{i})}{\partial {x}_{k}}=-\,{u}_{i}{\beta }_{j}-{{\phi }}_{ij}$$

where ${{\phi }}_{ij}={{\sigma }^{\prime} }_{ik}{\rho }{F}_{kj}/(2{\mu }{\tau })$ is the constitutive law defining the plastic deformation rate, *μ* is the shear modulus, ${{\sigma }^{\prime} }_{ij}={{\sigma }}_{ij}-{{\sigma }}_{kk}{{\delta }}_{ij}$ is the tensor of deviatoric stress, *δ*_*ij*_ is Kronecker delta, and τ is the relaxation time, the introduction of which makes the material deform in a way phenomenologically consistent with Maxwell solid model. Note that when *F* is mentioned in the system accounting for inelastic deformation (e.g. (8)), it refers to the elastic deformation gradient tensor in fact, rather than total deformation gradient tensor. Further, the relaxation time τ used can be determined from Favrie *et al*.^16^ as

9 $$\frac{1}{\tau }=\{\begin{array}{cc}\frac{1}{{\tau }_{0}}\frac{{|\sigma {\rm{^{\prime} }}|}^{2}-\frac{2}{3}{({\sigma }_{y})}^{2}}{{\mu }^{2}} & {\rm{i}}{\rm{f}}\,\,{f}_{VM}(\sigma ) > 0,\\ 0 & {\rm{i}}{\rm{f}}\,\,{f}_{VM}(\sigma )\le 0,\end{array}$$

where ${f}_{VM}(\sigma )={|\sigma ^{\prime} |}^{2}-\frac{2}{3}{({\sigma }_{y})}^{2}$ is the yield function of von Mises, σ_*y*_ is the yield limit and τ_0_ is a characteristic relaxation time. We make use of the Von Mises criterion for the actual processing of (8). That is, we decide whether the plasticity effect should be introduced in (8) or not according to the comparison results of the equivalent stress derived its prescribed value which can be found in Drumheller *et al*.^29^ and Steinberg^30^. In detail, if the equivalent stress is less than the prescribed value, the plasticity effect is ignored and the elastic model is used directly. On the contrary, the plasticity is introduced via source terms in the equations for elastic deformation gradient tensor *F*.

It is noted that in conjunction with the equations for the deformation gradient tensor F one has the mass continuity equation (2). Similar to the treatment by Barton^31^, we use the continuity equation (2) rather than the strain conversation equation for the deformation gradient component *ρF*_11_ for maintaining a fully determined system. Then the governing equations can be expressed in matrix form:

10 $$\frac{\partial {\bf{U}}}{\partial t}+\frac{\partial {{\bf{F}}}^{i}}{\partial {x}_{i}}=-\,{{\bf{S}}}_{C}-{{\bf{S}}}_{P},$$

where **U** is the vector formed by the conservative variables and **F**^*i*^ is the corresponding flux vector. The two terms, **S**_*C*_ and **S**_*p*_, on right hand side of (10), are associated with the constraints for the deformation gradient tensor and the plastic deformation, respectively. Here,

11 $${\bf{U}}=(\begin{array}{c}\rho {\bf{u}}\\ \rho \\ \rho {F}_{12}\\ \rho {F}_{13}\\ \vdots \\ \rho {F}_{33}\\ \rho {\rm{E}}\end{array}),\,\,\,{{\bf{F}}}^{i}=(\begin{array}{c}{u}_{i}\rho {\bf{u}}-\sigma {e}_{i}\\ \rho {u}_{i}\\ {u}_{i}\rho {F}_{12}-{u}_{1}\rho {F}_{i2}\\ {u}_{i}\rho {F}_{13}-{u}_{1}\rho {F}_{i3}\\ \vdots \\ {u}_{i}\rho {F}_{33}-{u}_{3}\rho {F}_{i3}\\ {u}_{i}\rho E-(\sigma u){e}_{i}\end{array}),\,\,\,{{\bf{S}}}_{C}=(\begin{array}{c}0\\ 0\\ {u}_{1}\frac{\partial \rho {F}_{j2}}{\partial {x}_{j}}\\ {u}_{1}\frac{\partial \rho {F}_{j3}}{\partial {x}_{j}}\\ \vdots \\ {u}_{3}\frac{\partial \rho {F}_{j3}}{\partial {x}_{j}}\\ 0\end{array}),\,\,\,{{\bf{S}}}_{P}=\frac{\rho }{2\mu \tau }(\begin{array}{c}0\\ 0\\ {\sigma ^{\prime} }_{1k}{F}_{k2}\\ {\sigma ^{\prime} }_{1k}{F}_{k3}\\ \vdots \\ {\sigma ^{\prime} }_{3k}{F}_{k3}\\ 0\end{array}),$$

where *e*_*i*_ are the Cartesian unit vectors. Let ${\bf{W}}=({\bf{u}},{F}^{T}{e}_{1},{F}^{T}{e}_{2},{F}^{T}{e}_{3},S)$ be the vector of primitive variables. Equation (10) is rewritten as a hyperbolic quasi-linear system

12 $$\frac{\partial {\bf{W}}}{\partial t}+{{\bf{A}}}^{k}\frac{\partial {\bf{W}}}{\partial {x}_{k}}=-\,{{\bf{S}}}_{P,QL},$$

where the Jacobian matrix ${{\bf{A}}}^{k}$ is given by

13 $${{\bf{A}}}^{k}=[\begin{array}{ccccc}{u}_{k}{\rm{I}} & -{A}^{k1} & -{A}^{k2} & -{A}^{k3} & -{B}^{k}\\ -{F}^{T}{D}_{k1} & {u}_{k}{\rm{I}} & 0 & 0 & 0\\ -{F}^{T}{D}_{k2} & 0 & {u}_{k}{\rm{I}} & 0 & 0\\ -{F}^{T}{D}_{k3} & 0 & 0 & {u}_{k}{\rm{I}} & 0\\ 0 & 0 & 0 & 0 & {u}_{k}\end{array}],$$

and the vector of source terms is

14 $${{\bf{S}}}_{P,QL}=\frac{1}{2\mu \tau }(\begin{array}{c}0\\ \sigma {^{\prime} }_{1k}{F}_{k1}\\ \sigma {^{\prime} }_{1k}{F}_{k2}\\ \sigma {^{\prime} }_{1k}{F}_{k3}\\ \vdots \\ \sigma {^{\prime} }_{3k}{F}_{k3}\\ -\frac{1}{\rho T}\sum _{i,k=1}^{3}\sigma {^{\prime} }_{ik}{\sigma }_{ik}\end{array}),$$

with $${A}_{ij}^{lm}=\frac{1}{\rho }\frac{\partial {\sigma }_{li}}{\partial {F}_{mj}}$$, $${B}_{i}^{l}=\frac{1}{\rho }\frac{\partial {\sigma }_{li}}{\partial S}$$ and $${D}_{ij}={e}_{i}\otimes {e}_{j}^{T}$$.

The equation of state used in this paper for the specifc internal energy *ε* is constructed by Miller *et al*.^13^

15 $$\varepsilon ({l}_{1},{l}_{3})=-\,{\int }_{{V}_{0}}^{V}p(V)dV+\frac{\mu }{2{\rho }_{0}}({l}_{1}-3{l}_{3}^{\mathrm{1/3}}),$$

where *ρ*_0_ is the density of the initially unstressed medium, *V* = 1/*ρ* is the specific volume, *p*(*V*) is the hydrostatic pressure (Wilkins *et al*.^10^) with the expression as

16 $$p(V)={p}_{01}(\eta -\mathrm{1)}+{p}_{02}{(\eta -\mathrm{1)}}^{2}+{p}_{03}{(\eta -\mathrm{1)}}^{3}GPa,\,\,\eta ={V}_{0}/V,$$

and *l*_1_, *l*_2_, *l*_3_ are the three independent invariants of the right Cauchy-Green strain tensor *C* = *F*^*T*^*F*:

17 $${l}_{1}=trC={C}_{11}+{C}_{22}+{C}_{33},\,\,\,{l}_{2}=\frac{1}{2}[(trC{)}^{2}-tr({C}^{2})],\,\,\,{l}_{3}={\rm{\det }}\,C\mathrm{.}$$

Further, the material density, stress tensor, specific internal energy *ε* and temperature *T* can be represented as functions of the variables mentioned above:

18 $$\rho ={\rho }_{0}/{\rm{\det }}\,F,\,\,\sigma =\rho \frac{\partial \varepsilon }{\partial F}{F}^{T}=2\rho F\frac{\partial \varepsilon }{\partial C}{F}^{T},\,\,\varepsilon =\varepsilon ({F}_{ij},S),\,\,T=\frac{\partial \varepsilon }{\partial S}\mathrm{.}$$

### Numerical scheme

To solve the Equation (10), a splitting strategy is used at each time step (Strang *et al*.^32^). Let *H*^Δ*t*^ be the solution operator corresponding to the homogeneous part and the compatibility part of the problem and *S*^Δ*t*^ be the operator describing the contribution of the plastic source step over the time step Δ*t*. Starting from the initial data U^*n*^, which is the vector of volume averaged conserved variables stored at the cell centres, the solution for the next time level is computed using operator splitting

19 $${{\rm{U}}}^{n+1}={S}^{\frac{1}{2}{\rm{\Delta }}t}{H}^{{\rm{\Delta }}t}{S}^{\frac{1}{2}{\rm{\Delta }}t}({{\rm{U}}}^{n}\mathrm{).}$$

The operator *H*^Δ*t*^ is derived based on the spatially unsplit method

20 $${H}^{{\rm{\Delta }}t}({{\rm{U}}}^{n})={{\rm{U}}}^{n}-{\rm{\Delta }}t(\frac{{F}_{i+\mathrm{1/2,}j,k}-{F}_{i-\mathrm{1/2,}j,k}}{{\rm{\Delta }}{x}_{1}}+\frac{{F}_{i,j+\mathrm{1/2,}k}-{F}_{i,j-\mathrm{1/2,}k}}{{\rm{\Delta }}{x}_{2}}+\frac{{F}_{i,j,k+\mathrm{1/2}}-{F}_{i,j,k-\mathrm{1/2}}}{{\rm{\Delta }}{x}_{3}}+{S}_{C}),$$

where *x* = (*x*_1_, *x*_2_, *x*_3_) is the spatial coordinates, Δ*x*_*i*_ is the grid spacing in the direction *x*_*i*_ and *F*_*i*±1/2,*j*,*k*_, *F*_i,*j*±1/2,*k*_, *F*_*i*,*j*,*k*±1/2_ are the numerical fluxes evaluated at the cell boundaries.

Each single-component region is given a boundary of “ghost cell” data, determined from a boundary condition, or obtained by a Modified Ghost Method (MGM). The single-component regions are then advanced in time with high-order Godunov methods i.e. piecewise parabolic method (PPM)^33–35^. The approach we use for elastic-plastic solid is basically identical the approach described in Zhang^25^. Multi-dimensional non-split method is implemented for PPM. The fluxes in (20) are constructed by approximate one-dimensional Riemann solvers in the direction orthogonal to the cell sides of the Cartesian mesh. For the sake of simplicity we report here the main ideas of the scheme. While to see the details of PPM reconstruction and PPM characteristic tracing, please refer to Miller *et al*.^33^.

#### HLLD Riemann Solver

**Figure 10:**
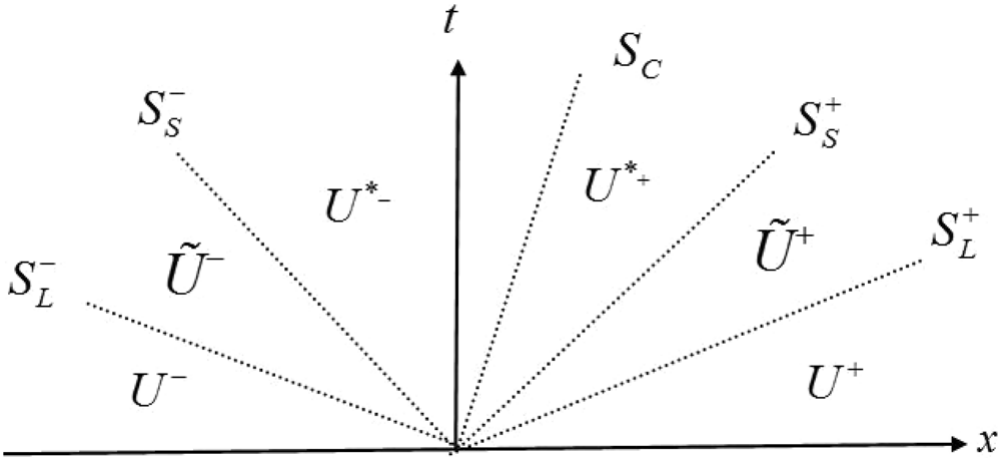
HLLD Riemann solver.

The main idea of HLLD Riemann solver that gives a nonlinear approximate solution is to assume a wave configuration for the solution that consists of five waves separating six constant states. The five waves include two slow waves, two fast waves and a contact discontinuity. Figure 10 shows four intermediate states: ${\tilde{{\bf{U}}}}^{-}$, U^*−^, U^*+^, and ${\tilde{{\bf{U}}}}^{+}$. We denote the fastest (longitudinal) waves between **U**^±^ and ${{\bf{U}}}^{\pm }$ as ${S}_{L}^{\pm }$ and the slow shear waves as ${S}_{S}^{\pm }$ that separate the states ${{\bf{U}}}^{\pm }$ and U^*±^. Each wave is considered to be a discontinuity and across each wave the Rankine-Hugoniot relation is satisfied:

21a $${S}_{L}^{\pm }{\tilde{{\bf{U}}}}^{\pm }-{\tilde{{\bf{F}}}}^{\pm }={S}_{L}^{\pm }{{\bf{U}}}^{\pm }-{{\bf{F}}}^{\pm }={{\bf{Q}}}_{L}^{\pm }$$

21b $${S}_{S}^{\pm }{\tilde{{\bf{U}}}}^{\pm }-{\tilde{{\bf{F}}}}^{\pm }={S}_{S}^{\pm }{{\bf{U}}}^{\ast \pm }-{{\bf{F}}}^{\ast \pm }={{\bf{Q}}}_{S}^{\pm }$$

21c $${S}_{C}{{\bf{U}}}^{\ast -}-{{\bf{F}}}^{\ast -}={S}_{C}{{\bf{U}}}^{\ast +}-{{\bf{F}}}^{\ast +}$$

Since there are more unknowns than equations based on (21a–c), we need to impose some other conditions. In order to obtain the unknown intermediate state vectors ${\tilde{{\bf{U}}}}^{\pm }$, ${{\bf{U}}}^{\ast \pm }$, ${\tilde{{\bf{F}}}}^{\pm }$ and ${{\bf{F}}}^{\ast \pm }$, we assume the following conditions are satisfied.

- Tangential velocities *u*_2_, *u*_3_ and tangential stresses σ_12_, σ_13_ are continuous across fast (longitudinal) waves and may jump across slow (shear) waves, while density *ρ*, normal velocity *u*_1_ and normal stress σ_11_ are just the opposite.
- Normal stress σ_11_ and normal velocity *u*_1_ are continuous across contact discontinuity; shear stress and tangential velocity are equal at the material interface for the ‘stick’ multi-material problem, while tangential component of the stress vector are zero for the ‘slip’ multi-material problem.

The left and right fastest wave speeds are computed as

22 $${S}_{L}^{-}=\,{\rm{\min }}({\lambda }_{1}({{\bf{W}}}_{L}),{\lambda }_{1}({{\bf{W}}}_{R}));\,\,\,{S}_{L}^{+}=\,{\rm{\max }}({\lambda }_{13}({{\bf{W}}}_{L}),{\lambda }_{13}({{\bf{W}}}_{R})),$$

and the slow wave speeds are estimated by

23 $${S}_{S}^{-}=\,{\rm{\min }}({\lambda }_{2}({\tilde{{\bf{W}}}}_{L}),{\lambda }_{2}({\tilde{{\bf{W}}}}_{R}));\,\,\,{S}_{S}^{+}=\,{\rm{\max }}({\lambda }_{12}({\tilde{{\bf{W}}}}_{L}),{\lambda }_{12}({\tilde{{\bf{W}}}}_{R}\mathrm{)).}$$

Within HLL approximation^20^, the intermediate wave speed *S*_*C*_ in the present solver is evaluated as

24 $$\begin{array}{rcl}{S}_{C} & = & \frac{({S}^{+}{\rho }^{+}{u}_{1}^{+}-{S}^{-}{\rho }^{-}{u}_{1}^{-})-({\rho }^{+}{u}_{1}^{+}{u}_{1}^{+}-{\sigma }_{11}^{+}-({\rho }^{-}{u}_{1}^{-}{u}_{1}^{-}-{\sigma }_{11}^{-}))}{({S}^{+}{\rho }^{+}-{S}^{-}{\rho }^{-})-({\rho }^{+}{u}_{1}^{+}-{\rho }^{-}{u}_{1}^{-})}\\  & = & \frac{{Q}_{L}^{+}\mathrm{(1)}-{Q}_{L}^{-}\mathrm{(1)}}{{Q}_{L}^{+}\mathrm{(4)}-{Q}_{L}^{-}\mathrm{(4)}},\end{array}$$

where ${Q}_{L}^{\pm }(i)$ and ${Q}_{R}^{\pm }(i)$ express the *i*-th components of the vectors ${Q}_{L}^{\pm }$ and ${Q}_{R}^{\pm }$, respectively. From the wave speeds above, $\tilde{{\bf{U}}}$ state can be described as:

25 $$\begin{array}{rcl}{\tilde{u}}_{1}^{\pm } & = & {S}_{C};\,{\tilde{u}}_{\mathrm{2,3}}^{\pm }={u}_{\mathrm{2,3}}^{\pm };\\ {\tilde{\rho }}^{\pm } & = & {\rho }^{\pm }\frac{{S}_{L}^{\pm }-{u}_{1}^{\pm }}{{S}_{L}^{\pm }-{S}_{C}}=\frac{{Q}_{L}^{\pm }\mathrm{(4)}}{{S}_{L}^{\pm }-{S}_{C}};\\ {\tilde{\sigma }}_{11}^{\pm } & = & {\rho }^{\pm }({S}_{L}^{\pm }-{u}_{1}^{\pm })({u}_{1}^{\pm }-{S}_{C})+{\sigma }_{11}^{\pm };{\tilde{\sigma }}_{12}^{\pm }={\sigma }_{12}^{\pm };{\tilde{\sigma }}_{13}^{\pm }={\sigma }_{13}^{\pm };\\ {\tilde{F}}_{1i}^{\pm } & = & ({\rho }^{\pm }{F}_{1i}^{\pm })/{\tilde{\rho }}^{\pm };{\tilde{F}}_{2i}^{\pm }={F}_{2i}^{\pm };{\tilde{F}}_{3i}^{\pm }={F}_{3i}^{\pm };\\ {\tilde{E}}^{\pm } & = & ({Q}_{L}^{\pm }\mathrm{(13)}-{{\tilde{u}}_{i}}^{\pm }{\tilde{\sigma }}_{1i}^{\pm })/{Q}_{L}^{\pm }\mathrm{(4).}\end{array}$$

Also, the intermediate states ${{\bf{U}}}^{\ast }$ are obtained as:

26 $${\rho }^{\ast \pm }={\tilde{\rho }}^{\pm };\,\,{u}_{1}^{\ast \pm }={\tilde{u}}_{1}^{\pm };\,\,{\sigma }_{11}^{\ast \pm }={\tilde{\sigma }}_{11}^{\pm }$$

For the ‘stick’ multi-material problem, the following formulas are satisfied:

27 $$\begin{array}{rcl}{u}_{2}^{\ast +} & = & {u}_{2}^{\ast -}=\frac{{\sigma }_{12}^{-}-{\sigma }_{12}^{+}+{\tilde{\rho }}^{-}{u}_{2}^{-}({S}_{S}^{-}-{S}_{C})-{\tilde{\rho }}^{+}{u}_{2}^{+}({S}_{S}^{+}-{S}_{C})}{{\tilde{\rho }}^{-}({S}_{S}^{-}-{S}_{C})-{\tilde{\rho }}^{+}({S}_{S}^{+}-{S}_{C})}=\frac{{Q}_{S}^{+}\mathrm{(2)}-{Q}_{S}^{-}\mathrm{(2)}}{{Q}_{S}^{+}\mathrm{(4)}-{Q}_{S}^{-}\mathrm{(4)}};\\ {u}_{3}^{\ast +} & = & {u}_{3}^{\ast -}=\frac{{\sigma }_{13}^{-}-{\sigma }_{13}^{+}+{\tilde{\rho }}^{-}{u}_{3}^{-}({S}_{S}^{-}-{S}_{C})-{\tilde{\rho }}^{+}{u}_{3}^{+}({S}_{S}^{+}-{S}_{C})}{{\tilde{\rho }}^{-}({S}_{S}^{-}-{S}_{C})-{\tilde{\rho }}^{+}({S}_{S}^{+}-{S}_{C})}=\frac{{Q}_{S}^{+}\mathrm{(3)}-{Q}_{S}^{-}\mathrm{(3)}}{{Q}_{S}^{+}\mathrm{(4)}-{Q}_{S}^{-}\mathrm{(4)}};\\ {\sigma }_{12}^{\ast \pm } & = & {Q}_{S}^{\pm }\mathrm{(2)}-{u}_{2}^{\ast \pm }{Q}_{S}^{\pm }\mathrm{(4);}\\ {\sigma }_{13}^{\ast \pm } & = & {Q}_{S}^{\pm }\mathrm{(3)}-{u}_{3}^{\ast \pm }{Q}_{S}^{\pm }\mathrm{(4).}\end{array}$$

For the ‘slip’ multi-material problem, we obtain

28 $$\begin{array}{c}{u}_{2}^{\ast \pm }={u}_{2}^{\pm }+{\sigma }_{12}^{\pm }/{Q}_{S}^{\pm }\mathrm{(4);}\,\,\,{u}_{3}^{\ast \pm }={u}_{3}^{\pm }+{\sigma }_{13}^{\pm }/{Q}_{S}^{\pm }\mathrm{(4);}\,\,\,{\sigma }_{12}^{\ast \pm }={\sigma }_{13}^{\ast \pm }=0.\end{array}$$

This expression could be used in the particular case of a solid-inviscid fluid or solid-vacuum interaction. Expressions of other variables are described as follows

29 $$\begin{array}{rcl}{F}_{1i}^{\ast \pm } & = & {\tilde{F}}_{1i}^{\pm };\,\,\,{F}_{21}^{\ast \pm }=({Q}_{S}^{\pm }\mathrm{(7)}-{(\rho {F}_{11}{u}_{2})}^{\ast \pm })/{Q}_{S}^{\pm }\mathrm{(4);}\\ {F}_{22}^{\ast \pm } & = & ({Q}_{S}^{\pm }\mathrm{(8)}-{(\rho {F}_{12}{u}_{2})}^{\ast \pm })/{Q}_{S}^{\pm }\mathrm{(4);}\,\,\,{F}_{23}^{\ast \pm }=({Q}_{S}^{\pm }\mathrm{(9)}-{(\rho {F}_{13}{u}_{2})}^{\ast \pm })/{Q}_{S}^{\pm }\mathrm{(4);}\\ {F}_{31}^{\ast \pm } & = & ({Q}_{S}^{\pm }\mathrm{(10)}-{(\rho {F}_{11}{u}_{3})}^{\ast \pm })/{Q}_{S}^{\pm }\mathrm{(4);}\,\,\,{F}_{32}^{\ast \pm }=({Q}_{S}^{\pm }\mathrm{(11)}-{(\rho {F}_{12}{u}_{3})}^{\ast \pm })/{Q}_{S}^{\pm }\mathrm{(4);}\\ {F}_{33}^{\ast \pm } & = & ({Q}_{S}^{\pm }\mathrm{(12)}-{(\rho {F}_{13}{u}_{3})}^{\ast \pm })/{Q}_{S}^{\pm }\mathrm{(4);}\,\,\,{E}^{\ast \pm }=({Q}_{S}^{\pm }\mathrm{(13)}-{{u}_{i}}^{\ast \pm }{\sigma }_{1i}^{\ast \pm })/{Q}_{S}^{\pm }\mathrm{(4).}\end{array}$$

In addition, the boundary condition at the interface within a single material is set to be ‘stick’. The HLLD fluxes ${\tilde{{\bf{F}}}}^{\pm }$ and ${{\bf{F}}}^{\ast \pm }$ for Godunov’s scheme are obtained as

30 $${{\bf{F}}}_{hlld}=\{\begin{array}{lll}{{\bf{F}}}^{-} & if & {S}_{L}^{-} > \mathrm{0,}\\ {\tilde{{\bf{F}}}}^{-} & if & {S}_{L}^{-}\le 0\le {S}_{S}^{-},\\ {{\bf{F}}}^{\ast -} & if & {S}_{S}^{-}\le 0\le {S}_{C},\\ {{\bf{F}}}^{\ast +} & if & {S}_{C}\le 0\le {S}_{S}^{+},\\ {\tilde{{\bf{F}}}}^{+} & if & {S}_{S}^{+}\le 0\le {S}_{L}^{+},\\ {{\bf{F}}}^{+} & if & {S}_{L}^{+} < 0.\end{array}$$

#### Plastic step

Contribution from plastic source terms is accounted for by solving the following ordinary differential equations (ODEs):

31 $$\frac{d\rho F}{dt}=-\frac{1}{2\mu \tau }\sigma ^{\prime} (\rho F)$$

If we use an explicit Euler scheme, the global time step will be dictated by a CFL condition. However, if this limit way for time step is considered and the strain rates are high enough, the timescales associated with the relaxation operator could be relatively small and hence the system of ODEs Equation (33) will become stiff. Thus, we use the following semi-implicit scheme to prevent the potential of stiff ODEs instead:

32 $${(\rho F)}^{n+1}={(\rho F)}^{\ast }\exp {(-\frac{{\rm{\Delta }}t}{2\mu \tau }\sigma ^{\prime} )}^{\ast },$$

where (ρ*F*)^*n*+1^ is the solution at time *t*^*n*+1^, the term σ′ is taken explicitly at the previous step t^*^ and the term *ρF* is chosen implicitly.

### Material interface evolution

#### The Level-Set Equation

The level-set algorithm is useful for interface tracking in multi-material problem. The central idea of the level-set technique is to represent the moving interface Γ(*t*) as the level set {*φ* = 0} of a function *φ*. At any moment *t*, *φ*(**x**, *t*) equals to zero on the front Γ(*t*) and we obtain the evolution equation

33 $$\frac{d{\phi }}{dt}=\frac{\partial {\phi }}{\partial t}+V\cdot {\rm{\Delta }}{\phi }=\mathrm{0,}$$

where $$V=\frac{d{\bf{x}}}{dt}$$.

In general, *φ*(**x**, *t*) will not satisfy the signed distance over time. Therefore, we need to adopt the reinitialization algorithm that transforms *φ*(**x**, *t*) to make it be the signed distance from location **x** to interface Γ(*t*). This transformation is achieved by solving the following equation

34 $$\{\begin{array}{c}{{\phi }}_{\tau }={\rm{sign}}({{\phi }}_{0})(1-|\nabla {\phi }|),\\ {\phi }({\bf{x}},\,0)={{\phi }}_{0},\end{array}$$

to steady state, which is the desired signed distance function.

The 5th-order weighted essentially non-oscillatory (WENO) scheme coupled with the 3rd-order Runge-Kutta time integration scheme is applied to solve Equation (35).

#### HLLD Multi-Material Riemann Solver

The solution procedure for multi-material problems is similar to HLLD Riemann solver for the single material problem except solid-solid or solid-vacuum boundary conditions at the material interface. The known states **W**_*L*_ = **W**(*x*_*i*_, *t*^*n*^) and **W**_*R*_ = **W**(*x*_*i*+1_, *t*^*n*^) passing an interface of two materials at time *t*^*n*^ are used to pose a multi-material Riemann problem. The fluxes on the left and right sides of the interface are given by

35 $${{\bf{F}}}_{i+\mathrm{1/2}}^{l}={{\bf{F}}}^{\ast -},{{\bf{F}}}_{i+\mathrm{1/2}}^{r}={{\bf{F}}}^{\ast +}\mathrm{.}$$

We define the states in the respective materials’ ghost cells as **W**_*i*+1_ = **W**_*i*+2_ = **W**_*i*+3_ = W^*−^ and **W**_*i*−2_ = W_*i*−1_ = **W**_*i*_ = W^*+^. We also adopt an entropy fix technique^36^ to suppress ‘heating errors’. In practical applications, the initial values of **W**_L_ and **W**_R_ are set to be W^*−^ and W^*+^, respectively. With these settings, about 10 iterations reaches the convergence precision.

#### Multi-dimensional implementation of MGFM

For multi-dimensional problems, the implementation of MGFM lies in the fact that the material interfaces are not perpendicular to the coordinate axis, but at a certain angle with them. Thus, it is required to extrapolate the relevant physical quantity g in the direction normal to the material interface by solving the following transport equation:

36 $${g}_{t}\pm \mathop{n}\limits^{\rightharpoonup }\cdot \nabla g=\mathrm{0,}$$

where $\,\mathop{n}\limits^{\rightharpoonup }=\frac{\nabla \varphi }{|\nabla \varphi |}$ is the unit vector in the direction normal to material interface. When we perform extrapolation from the region of *ϕ* < 0 to that of *ϕ* > 0, the ‘+’ sign is used in (38) and the values of physical quantities in the region of *ϕ* < −ε are kept; while we perform extrapolation from the region of *ϕ* > 0 to that of *ϕ* < 0, the ‘−’ sign is adopted and the values of physical quantities in the region of ϕ > ε are maintained. ε is generally set to 3Δ*x*. Then the extrapolation value for material 1 and the real value for material 2 coexist on the ghost meshes at the boundaries of material 1. In order to solve the problem of the angle between the material interface and coordinate axis, one coordinate axis could be rotated to the normal direction of material interface, leading to the transformation of multi dimensional problem to one dimensional problem with the following transformation relationship formula:

37 $${(\begin{array}{c}U\\ F\\ S\end{array})}^{ROT}=(\begin{array}{c}{R}^{ROT}U\\ {R}^{ROT}F\,{R}^{RO{T}^{T}}\\ S\end{array}),$$

where

38 $${R}^{ROT}={({\overrightarrow{x}}_{1},{\overrightarrow{x}}_{2},{\overrightarrow{x}}_{3})}^{T}$$

39a $${\overrightarrow{x}}_{1}={\bf{n}}=(\begin{array}{c}{n}_{1}\\ {n}_{2}\\ {n}_{3}\end{array})$$

39b $${\overrightarrow{x}}_{2}=\frac{1}{\sqrt{\mathrm{2(1}-{n}_{1}{n}_{2}-{n}_{1}{n}_{3}-{n}_{2}{n}_{3})}}(\begin{array}{c}{n}_{2}-{n}_{3}\\ {n}_{3}-{n}_{1}\\ {n}_{1}-{n}_{2}\end{array})$$

39c $${\overrightarrow{x}}_{3}=\frac{1}{\sqrt{\mathrm{2(1}-{n}_{1}{n}_{2}-{n}_{1}{n}_{3}-{n}_{2}{n}_{3})}}(\begin{array}{c}{n}_{1}({n}_{2}+{n}_{3})-{n}_{2}^{2}-{n}_{3}^{2}\\ {n}_{2}({n}_{1}+{n}_{3})-{n}_{1}^{2}-{n}_{3}^{2}\\ {n}_{3}({n}_{1}+{n}_{2})-{n}_{1}^{2}-{n}_{2}^{2}\end{array})$$

if |*n*_2_ + *n*_3_| ≤ |*n*_2_ − *n*_3_|, otherwise

40a $${\overrightarrow{x}}_{1}={\bf{n}}=(\begin{array}{c}{n}_{1}\\ {n}_{2}\\ {n}_{3}\end{array})$$

40b $${\overrightarrow{x}}_{2}=\frac{1}{\sqrt{\mathrm{2(1}+{n}_{3}({n}_{2}-{n}_{1})+{n}_{2}{n}_{1})}}(\begin{array}{c}{n}_{2}+{n}_{3}\\ {n}_{3}-{n}_{1}\\ -({n}_{1}+{n}_{2})\end{array})$$

40c $${\overrightarrow{x}}_{3}=\frac{1}{\sqrt{\mathrm{2(1}+{n}_{3}({n}_{2}-{n}_{1})+{n}_{2}{n}_{1})}}(\begin{array}{c}{n}_{1}({n}_{3}-{n}_{2})-{n}_{2}^{2}-{n}_{3}^{2}\\ {n}_{2}({n}_{1}+{n}_{3})+{n}_{1}^{2}+{n}_{3}^{2}\\ {n}_{3}({n}_{1}-{n}_{2})-{n}_{1}^{2}-{n}_{2}^{2}\end{array})$$

After the solution process of the transformed one-dimensional problem, the coordinate axis and all physical quantities should be transformed back to the original coordinate system.

In summary, the MGM solving procedures for the Riemann problem with multi materials are as follows:

- judge the locations of material interfaces according to the level-set field;
- extrapolate the values of physical quantities on real meshes to ghost meshes at material boundaries for each material;
- determine the physical quantities at ghost meshes at each material boundary by solving Riemann problem with multi materials with the use of HLLD solver for multi material case;
- update the status of each material to next time step by using the above solution method for single material;
- update the level-set field to the next time level and reinitialize the level-set field;
- repeat steps (a–e) until the desired time-level is reached.

## References

[1] Howell BP, Ball GJ (2002). A free-lagrange augmented godunov method for the simulation of elastic-plastic solids. Journal of Computational Physics.

[2] Cheng J-B, Toro EF, Jiang S, Yu M, Tang W (2015). A high-order cell-centered lagrangian scheme for one-dimensional elastic–plastic problems. Computers & Fluids.

[3] Fridrich D, Liska R, Wendroff B (2017). Cell-centered lagrangian lax-wendroff hll hybrid method for elasto-plastic flows. Computers & Fluids.

[4] Brauer AD (2017). A cartesian scheme for compressible multimaterial hyperelastic models with plasticity. Communications in Computational Physics.

[5] Ghaisas, N. S., Subramaniam, A. & Lele, S. K. High-order eulerian methods for elastic-plastic flow in solids and coupling with fluid flows. In *Aiaa Fluid Dynamics Conference* (2016).

[6] Hill DJ, Pullin D, Ortiz M, Meiron D (2010). An eulerian hybrid weno centered-difference solver for elastic–plastic solids. Journal of Computational Physics.

[7] Bertram, A. Elasticity and plasticity of large deformations (Springer, 2005).

[8] Naghdi PM (1990). A critical review of the state of finite plasticity. J. Appl. Math. Phys (ZAMP).

[9] Romenskii E (1974). Hypoelastic form of equations in nonlinear elasticity theory. Journal of Applied Mechanics and Technical Physics.

[10] Wilkins, M. L. Calculation of elastic-plastic flow. Tech. Rep., DTIC Document (1963).

[11] Kluth G, Despres B (2008). Perfect plasticity and hyperelastic models for isotropic materials. Continuum Mechanics & Thermodynamics.

[12] Plohr, B. J. & Sharp, D. H. *A conservative formulation for plasticity* (Academic Press, Inc., 1992).

[13] Miller G, Colella P (2001). A high-order eulerian godunov method for elastic–plastic flow in solids. Journal of computational physics.

[14] Barton P, Drikakis D, Romenski E (2010). An eulerian finite-volume scheme for large elastoplastic deformations in solids. International journal for numerical methods in engineering.

[15] Hank S, Gavrilyuk S, Favrie N, Massoni J (2017). Impact simulation by an eulerian model for interaction of multiple elastic-plastic solids and fluids. International Journal of Impact Engineering.

[16] Favrie N, Gavrilyuk S (2011). Dynamics of shock waves in elastic-plastic solids. Esaim Proceedings.

[17] Colella P, Woodward PR (1984). The piecewise parabolic method (ppm) for gas-dynamical simulations. Journal of computational physics.

[18] Roe PL (1981). Approximate riemann solvers, parameter vectors, and difference schemes. Journal of Computational Physics.

[19] Titarev V, Romenski E, Toro E (2008). Musta-type upwind fluxes for non-linear elasticity. International journal for numerical methods in engineering.

[20] Harten A, Lax PD (1983). & Leer, B. v. On upstream differencing and godunov-type schemes for hyperbolic conservation laws. SIAM review.

[21] Batten P, Clarke N, Lambert C, Causon DM (1997). On the choice of wavespeeds for the hllc riemann solver. Siam Journal on Scientific Computing.

[22] Toro EF, Spruce M, Speares W (1994). Restoration of the contact surface in the hll-riemann solver. Shock Waves.

[23] Ndanou S, Favrie N, Gavrilyuk S (2015). Multi-solid and multi-fluid diffuse interface model: Applications to dynamic fracture and fragmentation. Journal of Computational Physics.

[24] Cheng J (2016). Harten-lax-van leer-contact (hllc) approximation riemann solver with elastic waves for one-dimensional elastic-plastic problems. Applied Mathematics and Mechanics -English Edition.

[25] Zhang W (2017). The piecewise parabolic method for riemann problems in nonlinear elasticity. Scientific Reports.

[26] Maire P-H, Abgrall R, Breil J, Loubere R, Rebourcet B (2013). A nominally second-order cell-centered lagrangian scheme for simulating elastic-plastic flows on two-dimensional unstructured grids. Journal of Computational Physics.

[27] Godunov S, Romenskii E (1972). Nonstationary equations of nonlinear elasticity theory in eulerian coordinates. Journal of Applied Mechanics and Technical Physics.

[28] Godunov, S. K. & Romenskii, E. *Elements of continuum mechanics and conservation laws* (Springer Science & Business Media, 2003).

[29] Drumheller DS, Harris JG (2002). Introduction to wave propagation in nonlinear fluids and solids. Journal of the Acoustical Society of America.

[30] Steinberg DJ, Lund CM (1989). A constitutive model for strain rates from 10-4 to 106 s-1. Journal of Applied Physics.

[31] Barton PT, Drikakis D, Romenski E, Titarev VA (2009). Exact and approximate solutions of riemann problems in non-linear elasticity. Journal of Computational Physics.

[32] Strang G (1968). On the construction and comparison of difference schemes 5. Siam Journal on Numerical Analysis.

[33] Miller G, Colella P (2002). A conservative three-dimensional eulerian method for coupled solid–fluid shock capturing. Journal of Computational Physics.

[34] Stone JM, Gardiner TA, Teuben P, Hawley JF, Simon JB (2008). Athena: A new code for astrophysical mhd. Astrophysical Journal Supplement.

[35] Zheng J, Lee T (2013). A high-resolution method for compressible two-fluid flows and simulation of three-dimensional shock–bubble interactions. International Journal for Numerical Methods in Fluids.

[36] Liu T, Khoo B, Yeo K (2003). Ghost fluid method for strong shock impacting on material interface. Journal of computational physics.

